# Research progress of CRISPR-based biosensors and bioassays for molecular diagnosis

**DOI:** 10.3389/fbioe.2022.986233

**Published:** 2022-09-16

**Authors:** Kun Chen, Ziyi Shen, Guanzhen Wang, Wei Gu, Shengchao Zhao, Zihan Lin, Wei Liu, Yi Cai, Gohar Mushtaq, Jia Jia, Chunpeng (Craig) Wan, Tingdong Yan

**Affiliations:** ^1^ School of Life Sciences, Shanghai University, Shanghai, China; ^2^ University and College Key Lab of Natural Product Chemistry and Application in Xinjiang, School of Chemistry and Environmental Science, Yili Normal University, Yining, China; ^3^ Key Laboratory of Molecular Target & Clinical Pharmacology and The State & NMPA Key Laboratory of Respiratory Disease, School of Pharmaceutical Sciences and The Fifth Affiliated Hospital, Guangzhou Medical University, Guangzhou, China; ^4^ Center for Scientific Research, Faculty of Medicine, Idlib University, Idlib, Syria; ^5^ Jiangxi Key Laboratory for Postharvest Technology and Nondestructive Testing of Fruits and Vegetables, College of Agronomy, Jiangxi Agricultural University, Nanchang, China

**Keywords:** molecular diagnostics, CRISPR/Cas, biosensor, non-nucleic-acid analytes, nucleic acid detection

## Abstract

CRISPR/Cas technology originated from the immune mechanism of archaea and bacteria and was awarded the Nobel Prize in Chemistry in 2020 for its success in gene editing. Molecular diagnostics is highly valued globally for its development as a new generation of diagnostic technology. An increasing number of studies have shown that CRISPR/Cas technology can be integrated with biosensors and bioassays for molecular diagnostics. CRISPR-based detection has attracted much attention as highly specific and sensitive sensors with easily programmable and device-independent capabilities. The nucleic acid-based detection approach is one of the most sensitive and specific diagnostic methods. With further research, it holds promise for detecting other biomarkers such as small molecules and proteins. Therefore, it is worthwhile to explore the prospects of CRISPR technology in biosensing and summarize its application strategies in molecular diagnostics. This review provides a synopsis of CRISPR biosensing strategies and recent advances from nucleic acids to other non-nucleic small molecules or analytes such as proteins and presents the challenges and perspectives of CRISPR biosensors and bioassays.

## 1 Introduction

Accurate, rapidly available, and highly sensitive detection tools are essential to human health. In diagnosing diseases, early detection is significant for effective disease treatment. Early intervention can reduce the duration of untreated illness, slow down the disease progression and control the spread of infectious diseases (Parolo and Merkoci, 2013). One example is the ongoing worldwide epidemic of COVID-19, for which an increasing number of tests are being developed to detect nucleic acid markers of SARS-CoV-2 (Rahimi et al., 2021). In the field of food safety, fast detection of toxic substances in food allows for monitoring the quality and safety from raw material production to processing to packaging (Mao et al., 2022a). In addition, wearable devices with detection capabilities are being explored, with features such as easy sampling, high speed of detection, and easy reading of results (Nguyen et al., 2021), which provide a prototype for future out-of-lab testing.

Clustered, regularly interspaced short palindromic repeats (CRISPR) system was first discovered in 1987 (Ishino et al., 1987). CRISPR is a sequence of genes found in many bacteria and most archaea (Mojica et al., 2000). The CRISPR system is a defensive mechanism containing the CRISPR gene and the CRISPR-associated sequence proteins (Cas protein), which is an enzyme with the ability to cleave nucleic acids, relying on the guidance of RNA to target regions thereby defending against invasion by exogenous genes (Sander and Joung, 2014). These significant discoveries have provided new ideas and avenues for expanding the gene-editing tools (Cong et al., 2013).

In recent years, the classification of CRISPR-Cas proteins has gained increasing interest and attention as more Cas enzymes have been discovered. It has been reported that a multivariate classification system based on phylogenetic, comparative genomics, and structural analysis has been established (Makarova et al., 2011). Subsequently, Cas proteins have been divided into two class systems, Class I proteins, which have multiple subunits with CRISPR RNA (crRNA) complexes, and Class II, which rely on a single protein to function (Makarova et al., 2015). To date, the four Cas enzymes commonly used in nucleic acid detection all originate from the Class II system, which is capable of differential activity on single-stranded DNA (ssDNA), double-stranded DNA (dsDNA), and single-stranded RNA (ssRNA), respectively. Cas9 protein has a cis-cleavage function, recognizing the protospacer adjacent motif (PAM) sequence that binds specifically to its guide RNA, and efficient cleavage occurs. Cas12, Cas13, and Cas14 (Cas12f) all have a trans cleavage function in addition to cis cleavage, namely the ability to cleave non-selective collateral nucleic acids (Aman et al., 2020).

The CRISPR system-based assays are specific, programmable, and accessible. Further improvements have been reported for representative platforms targeting nucleic acid material, such as specific high sensitivity enzymatic reporter unlocking (SHERLOCK) and DNA endonuclease-targeted CRISPR *trans-*reporter (DETECTR), as well as more advanced platforms inspired by such platforms (Bonini et al., 2021). In previous reviews, the potential strengths of some platforms for nucleic acid detection and disease diagnostic applications have been summarized. However, some tools have recently been updated, with a growing number of analyses for non-nucleic acid material expanding the range of CRISPR-based tools, leading to significant advances in other areas of human health. Therefore, those newly evolved methods must be sorted out to illustrate the ongoing development and future trends. This review provides an overview of detection strategies ranging from nucleic acid to non-nucleic-acid materials based on improved platforms and an outlook on applying CRISPR system detection (Supplementary Material S1) in practical challenge scenarios such as disease diagnosis and food safety.

## 2 CRISPR-based biosensors and bioassays

### 2.1 CRISPR system

In archaea and bacteria, the CRISPR system functions by targeting nucleic acids to achieve immunity against foreign invasion (Barrangou et al., 2007). Although different CRISPR-Cas systems are found among them, crRNA is the core of their target molecules (Brouns et al., 2008). The editing of crRNA-employed genetic engineering allows the function of Cas proteins that hybridize with specific nucleic acid sequences (Cong et al., 2013). This exemplifies the programmability, simplicity, and high sensitivity of CRISPR, demonstrating the power of using CRISPR for detection.

Cas9 (part of Class II, Type II system) produces complementary trans-activating crRNA (tracrRNA) utilizing pre-crRNA repeat sequences for maturation (Jinek et al., 2012). In the construction of detection tools, tracrRNA is typically constructed in combination with crRNA as a small-guided-RNA (sgRNA), with its hybridized sequences limited to PAM or protospacer flanking sequences. Cas9 has only a cis-cleavage function and is capable of causing dsDNA cleavage (Chen and Doudna, 2017). Cleavage activity for ssDNA and ssRNA has also been reported in the presence of PAM-presenting oligonucleotides (PAMmers) sequence designed to complement the target sequence (O’Connell et al., 2014; Sternberg et al., 2014). This property has been successfully used in coupling with real-time fluorescence analysis modalities for the detection of the specific nucleic acid target (Zhou et al., 2018).

Cas12 belongs to the Class II, Type V system, with Cas12a being one of its widely used subtypes. Cas12 recognizes complementary ssDNA or dsDNA with a PAM sequence under the guidance of its crRNA, directing the cleavage of DNA through a single RuvC structural domain (Zetsche et al., 2015). Its family has another commendable category of nucleases, Cas12f (formerly Cas14) in the latest classification system (Makarova et al., 2020), which is less than half the size of Cas9, has promising ssDNA cleavage activity, and can be generated without the need for PAM sequences (Harrington et al., 2018; Karvelis et al., 2020). Cas12 has been reported to have efficient trans cleavage activity. It can induce robust and nonspecific ssDNA cleavage when it cleaves dsDNA in a sequence-specific manner, a property that has been widely used in assays since its discovery in 2018 (Li et al., 2018a; Chen et al., 2018).

Cas13, which belongs to the Class II, Type VI system, contains the HEPN structural domain that binds to RNA for cleavage and is guided by a single RNA (Abudayyeh et al., 2017). Cas13a (formerly C2c2) is one of its widely used subtypes. It has been found that the Cas13a-crRNA complex, once activated by binding to its target RNA, cleaves it as well as other non-specific RNAs. This trans cleavage capability expands the range of targets to be detected (Abudayyeh et al., 2016).

### 2.2 Readout system

#### 2.2.1 Colorimetric technique

**FIGURE 1:**
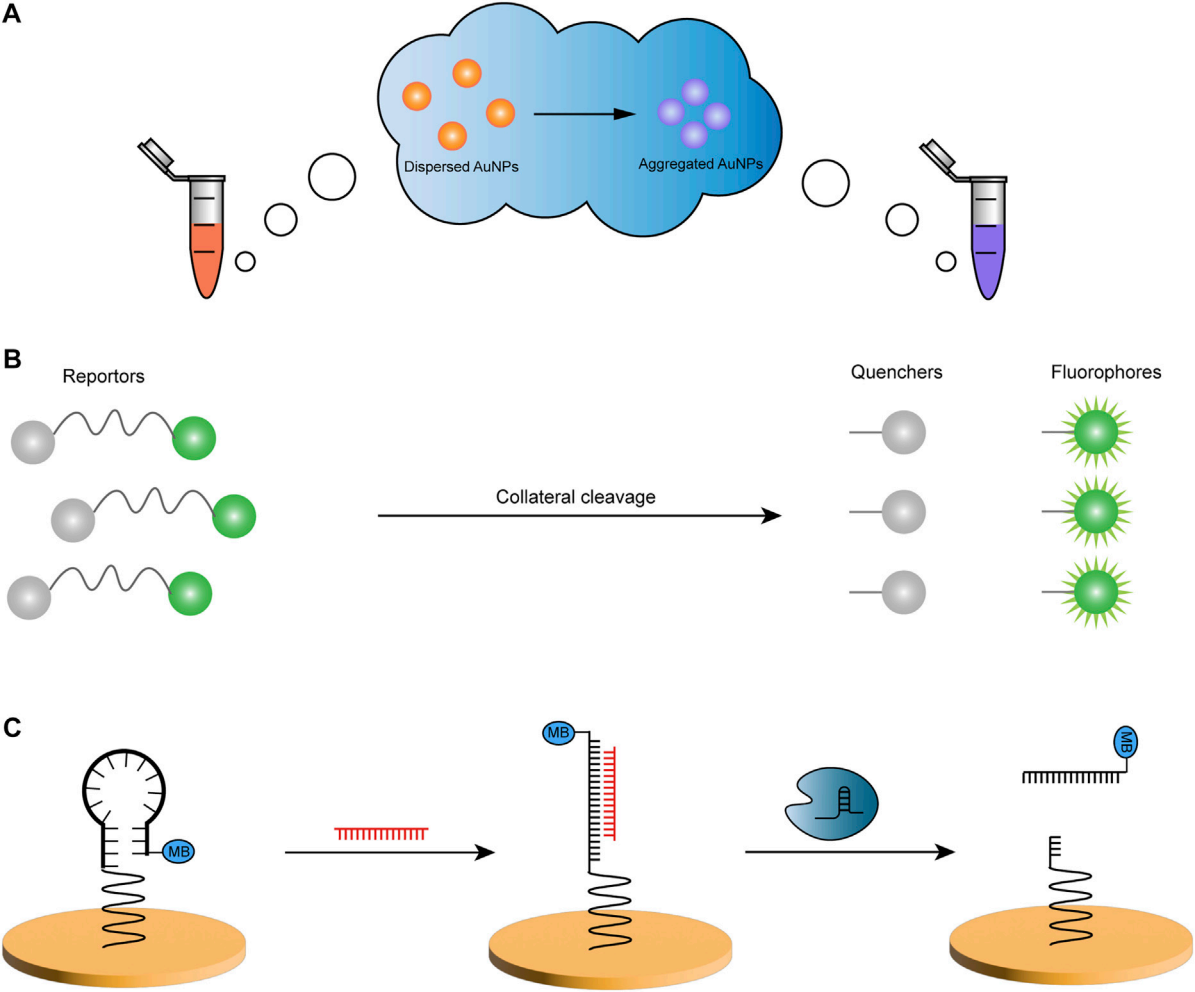
Examples of three common readout methods. **(A)** The color of the solution in the centrifuge tube changes due to the aggregation of AuNPs. **(B)** The reporter consists of a fluorophore and a quencher, linked by a nucleic acid molecule. After its collateral cleavage by the CRISPR system, the change in the distance leads to the generation of fluorescent energy. **(C)** The conformation of E-DNA changes from hairpin to linear dsDNA when the ssDNA of the gold electrode hairpin structure is complementary to the target ssDNA (red), and Cas12a cleaves the linear DNA to release the MB strand. MB, methylene blue.

Colorimetric methods analyze the content of substances by comparing the shades of light in colored solutions and have unique readout systems in biosensing owing to their rapid, intuitive, and low-cost characteristics. Among the standard detection probes used in colorimetric systems, gold nanoparticles (AuNPs) are frequently used. They have particular optical properties such as high extinction coefficient, localized surface plasmon resonance (LSPR), and inherent photostability (Aldewachi et al., 2018). LSPR features lead to color changes in AuNP and are utilized to construct CRISPR-based colorimetric sensors (Figure 1A), as physiological processes and biomolecular interactions affect the dispersion and aggregation of AuNPs (Baptista et al., 2008).

However, colorimetric sensors also suffer from the same problems of false positives and negatives caused by the instability of the reporter molecule or due to the weak signal (Xie et al., 2021). In contrast to the other methods, the naked-eye readout system requires a stable optical signal, and the procedure does not rely on sophisticated equipment. Still, it can be a complex and time-consuming process. In addition, non-specific interference is a significant challenge for the colorimetric method, and refinements to AuNPs are continually required to improve the accuracy of their reporting signals (Xu et al., 2021).

#### 2.2.2 Fluorescence and luminescence technique

In disease diagnosis, fluorescence technology is widely used in bioassays (Yue et al., 2018; Lu Y. et al., 2022). The fluorescence resonance energy transfer (FRET) sensor has the advantages of superb sensitivity, high specificity, and fast responsiveness (Zhang et al., 2019). FRET is a process in which non-radiative energy is transferred from donor fluorophore to acceptor chromophore, where the donor is electronically excited, with the acceptor both being photosensitive molecules (Zhang et al., 2019; Phan et al., 2022). In previous strategies, a pair of closely positioned donors and acceptors is usually designed to excite a reporter signal using specific recognition of the target molecule by the acceptor and conformational changes of the FRET system (Zhang et al., 2019). Quenchers are used with increasing frequency in acceptors due to their broad absorption spectra and high extinction coefficients and for the construction of detection systems containing pairs of fluorophore and quenchers of molecules linked by DNA or RNA probes (Figure 1B) (Phan et al., 2022). A CRISPR-based sensor using FRET technology would utilize the trans cleavage activity of the Cas enzyme to digest the probe by specific recognition followed by excitation of the fluorescent signal (Phan et al., 2022).

In addition to FRET, researchers have similarly focused on luminescent resonance energy transfer (LRET) due to its more significant advantages by compensating for the shortcomings of FRET, for instance, micelles and liposomes scatter the light which complicates the FRET applications in membrane-protein research (Sohail et al., 2022). LRET uses luminescent lanthanide chelates as donors with millisecond lifetime, compared to FRET which uses a donor with millisecond lifetime. In addition to longer lifetime, LRET also has the advantage of using multiple acceptors with flexibility, which is better for detecting conformational changes and dynamics of proteins or other macromolecules (Dolino et al., 2014). In the application of integrated CRISPR sensing, Li established an LRET-based functional DNA (fDNA)-regulated CRISPR/Cas12a biosensor by combining holographic optical tweezers with energy-concentrating upconversion luminescence nanoparticle boosting enhancement of LRET (Li C.-Y. et al., 2021). This has a more significant advantage in detecting non-nucleic acid analytes since the approach improves sensitivity and imaging capability (Sohail et al., 2022).

#### 2.2.3 Electrochemical technique

Electrochemical biosensors can convert biochemical reactions into electrical signals that can be measured with simplicity, low cost, and high selectivity (Fritea et al., 2018). It has been combined with CRISPR to develop fast and less equipment-dependent detection tools through the use of membranes or microfluidics on working electrodes in association with different materials to eliminate interference and amplify signals (Bruch et al., 2019; Bruch et al., 2021; Phan et al., 2022), such as electrochemical DNA (E-DNA) sensors (Figure 1C) (Dai et al., 2019; Xu et al., 2020). In addition to the aforementioned fluorescence and luminescence techniques alone for bioanalysis, luminescence methods have also been exploited by integrating them with electrochemical techniques to create new electrochemiluminescent (ECL) biosensors. They work by generating substances on the electrode surface that change from stable precursors to unstable intermediates and consequently form luminescent excited states *via* electron transfer involving the conversion of electrical and radiative energy (Richter, 2004). ECL improves on conventional electrochemical sensors with high sensitivity, low background interference, electrochemical controllability, and excellent selectivity (He et al., 2020), which can build a more stable bioassay platform and play an essential role in the field of medical examinations (Qin et al., 2021; Suea-Ngam et al., 2021; Zhang et al., 2021).

### 2.3 Nucleic acid detection strategies

#### 2.3.1 Cas9-based biosensors and bioassays

**FIGURE 2:**
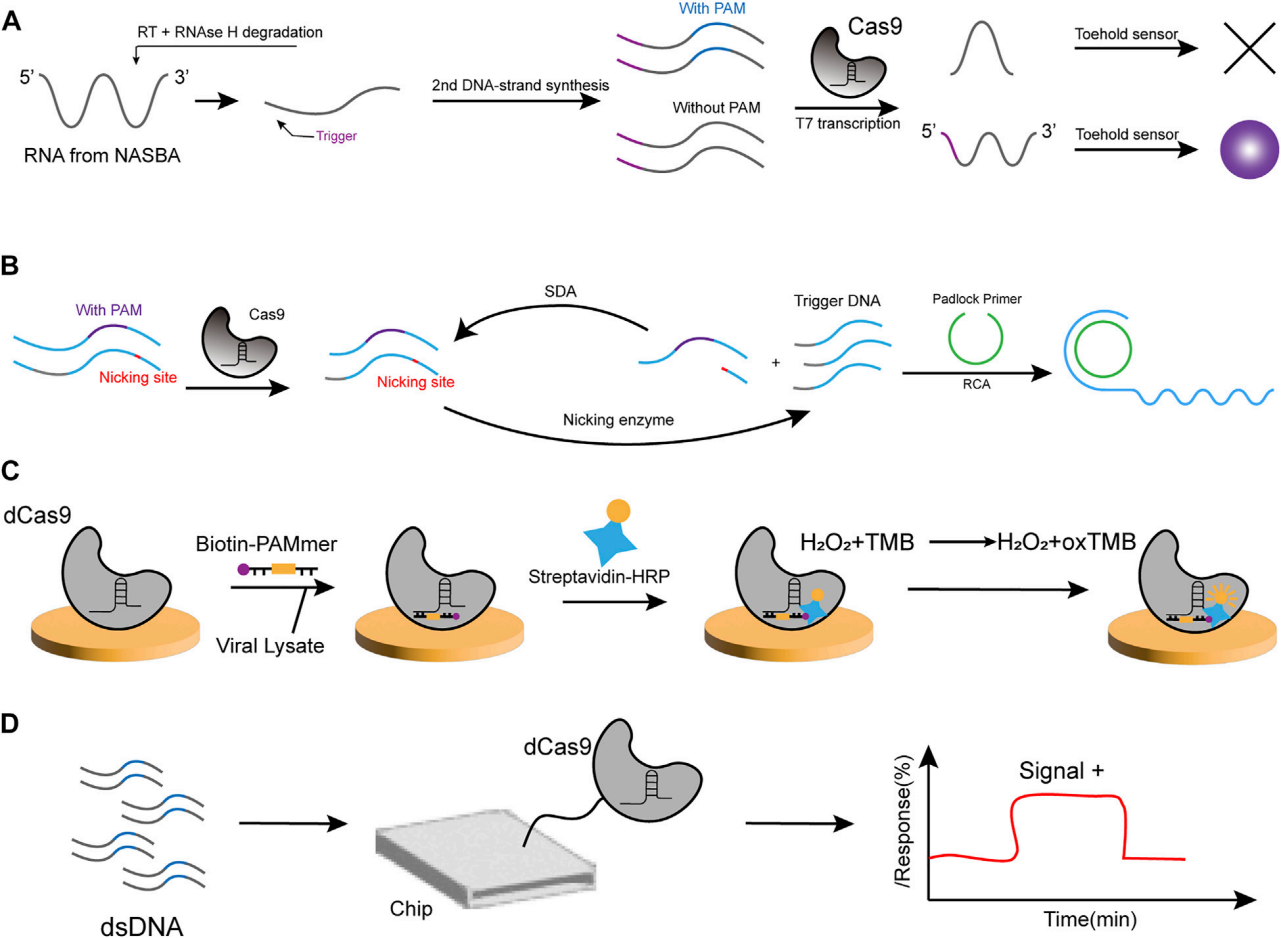
Strategies for Cas9-based nucleic acid sensor. **(A)** NASBA amplifies the RNA target, and the trigger sequence for the toehold switch is introduced. Subsequently, the RNA and DNA hybridization produces RNA that is degraded by RNAse H. The second DNA strand is synthesized, and it carries the T7 promoter sequence. The transcribed RNA can be used as starting material for NASBA and interact with the toehold switch. In the presence of PAM sequences, the RNA target site is shorter due to the cleaved template and cannot activate the sensor, and vice versa, producing a significant color change. **(B)** In the action of Cas9, broken dsDNA at the nicking site is further recognized by the nicking enzyme. Once the DNA polymerase reaches the nicking site, a new strand can be generated and the downstream strand is replaced. As a result, many short ssDNAs are generated to trigger rolling circle amplification (RCA). Subsequently, the RCA product can bind to oligonucleotide-spliced AuNPs, inducing the aggregation of AuNPs. SDA, Strand-displacement amplification. **(C)** Viral lysate and biotin-PAMmer are added to microplates immobilized with dCas9/gRNA complexes. Following, Streptavidin-HPR and TMB substrate solutions were added to the microplate. Finally, the yellow color is observed in the presence of the virus. **(D)** The CRISPR-chip is composed of a gFET structure with a complex of sgRNA and dCas9 formed on the graphene surface. When the target DNA is detected, dCas9 binds to the target DNA, which modulates the electrical properties of the gFET and leads to an electrical signal output. gEFT: graphene-based field-effect transistor.

In 2016, Collins et al. applied CRISPR/Cas technology to nucleic acid detection for the first time, successfully incorporating Cas9 protein to develop a paper-based sensor for the detection of Zika virus (ZIKV) (Pardee et al., 2016). In Collins’ study, they used an isothermal RNA amplification technique called NASBA (nucleic acid sequence-based amplification), where the first step of the process is the reverse transcription of the target RNA by a complementary reverse primer, thereby producing an RNA/DNA double-stranded body, followed by degradation of the RNA template in the presence of RNase H. Then, the T7 promoter containing the forward primer binds and initiates the extension of the complementary strand to produce a dsDNA product, which is finally transcribed into copies of the target RNA sequence. The reaction is carried out in an isothermal environment at 41°C after initial heating at 65°C. Once the nucleic acid amplification step is complete, the detection part is accomplished by a toehold switch, allowing the hairpin structure to release after the amplification product is added to the paper-based sensor. This would activate the synthesis of Lac Z, which reports by the colorimetric change of the yellow substrate into the red product. Due to the naturally occurring PAM sequence specificity of Cas9 protein, the NASBA-CRISPR cut (NASBACC) strategy (Figure 2A) was used for single-base recognition, enabling effective classification of different ZIKV strains while controlling the assay time to 3 h.

Another material for the Cas9 detection strategy is AuNP, also by utilizing colorimetric methods. Liu et al. constructed an assay based on the CRISPR/Cas9 system that triggered signal amplification to identify the *Phytophthora infestans* genomic DNA (Chang et al., 2019). Oligonucleotide-functionalized AuNPs hybridize with Cas9/sgRNA digestion products that have been isothermally amplified, resulting in a color change from burgundy to purple after aggregation. Strand-displacement amplification (SDA) reactions and rolling circle amplification (RCA) reactions occur here. First, the cleaved dsDNA generates new strands in the presence of DNA polymerase, which further generates many short ssDNA to trigger RCA (Figure 2B). After the RCA reaction, the product is hybridized with oligonucleotide-functionalized AuNPs, thus causing the changes. The method has a visual detection limit of 2 pM DNA and shows an excellent linear relationship between the ratio of A 650 nm/A 525 nm and model DNA concentrations of 0.2 pM to 20 nM, with good sensitivity and high specificity.

Kang’s team designed a (CRISPR)/Cas9 endonuclease dead (dCas9)-based colorimetric sensor to detect viruses such as SARS-CoV-2. They attached biotin to a PAMmer and achieved color change by (HRP)/3,3′,5,5′-tetramethylbenzidine (TMB) reaction, thereby effectively reducing the assay time (Figure 2C) (Moon et al., 2020). The dCas9 is a catalytically inactive enzyme produced by mutations in the RuvC and HNH structural domains of wild-type SpCas9. Although it has lost its gene-editing function, the retained gRNA binding ability makes it a powerful tool in many fields, including disease diagnosis (Kanafi and Tavallaei, 2022). For example, the Paired dCas9 (PC) reporter system was designed (Zhang et al., 2017) and used the split luciferase system to construct two dCas9-based sensors that recognize two fragments of the same DNA sequence, each with half of the firefly luciferase enzyme attached to the N- and C-terminal, respectively. When specific recognition is achieved, the integral enzyme catalyzes the bioluminescent reaction and reportedly detects analytes at concentrations ranging from 5 × 10^−5^ to 6 × 10^−3^ nmol/ml.

Li et al. developed a CRISPR/Cas9-triggered hairpin probe-mediated biosensing method (CHP) that differentially hybridizes long-stranded RCA products with fluorophore quencher-labeled molecular beacons, enabling real-time fluorescent readout (Wang et al., 2021). The authors found that CHP is highly specific for detecting circulating tumor DNA (ctDNA) in human serum without requiring DNA extraction and purification. Additionally, to improve the detection efficiency, AuNPs have been used to develop lateral flow nucleic acid detection tools capable of accurately detecting viruses (Wang X. et al., 2020; Xiong et al., 2021). Zhang et al. developed a way to use engineered crRNAs introduced into DNA (as enzymes or probes) to bind fluorescent reporter groups for testing (Safdar et al., 2022).

Cas9 has also been used for electrochemical methods of the assay. Another assay using dCas9 is named CRISPR-Chip, which immobilizes the dCas9/CrRNA complex on a graphene-based field-effect transistor (gEFT) to construct a sensor that detects the variable currents caused by changes in graphene conductivity and does not require a reporter molecule for the deletion in the dystrophin genes of Duchenne muscular dystrophy patients (Figure 2D) (Hajian et al., 2019). Similarly, the team reported the CRISPR-SNP-Chip system in order to achieve typing of single-nucleotide polymorphisms (SNPs) to escape the dependence on advanced sequencing technologies (Balderston et al., 2021). Liu et al. explored a primer-exchange-reaction (PER)-based biochemical circuit cascade using a Cas9 enzyme with only single-stranded cleavage activity. Driving a pair with different sgRNAs of Cas9 generates dsDNA with sticky ends, ligated to a hairpin-structure substrate, which subsequently generates a large number of short fragments that report the electrical signal of the detected DNA (Dai et al., 2020). Huang’s team developed a dCas9-ECL probe for sensitive and single-base specific DNA detection with good resistance to nonspecific interference and a low false positive detection rate (Wu L. et al., 2022). This shows that electrochemical biosensors can modularize the circuitry with better programmability.

#### 2.3.2 Cas12-based biosensors and bioassays

**FIGURE 3:**
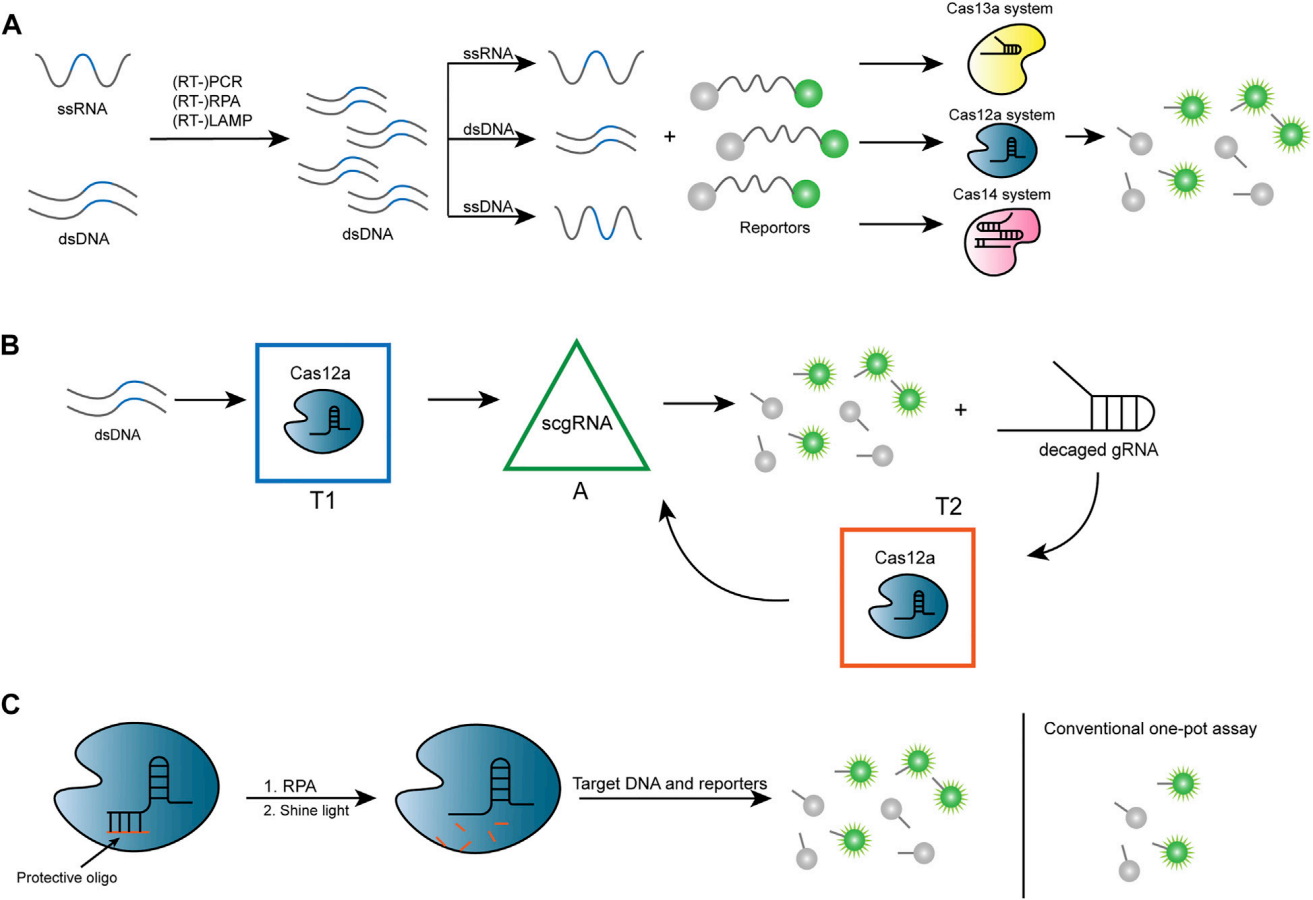
Strategies for Cas12 and Cas13-based nucleic acid sensor. **(A)** After amplification, ssRNA or dsDNA forms dsDNA and produces ssRNA, dsDNA, and ssRNA, depending on the different systems. After being mixed with fluorescent reporters, trans cleavage occurs through specific binding of different CRISPR/Cas systems, which subsequently generates a fluorescent signal. **(B)** In CONAN, there are three signal processors, namely transducer 1 (T1), transducer 2 (T2), and signal amplifier (A), where the parallel connection of A and T2 forms a positive feedback circuit and T1 is connected in series with the positive feedback circuit. Cas12a and gRNA for target dsDNA (gRNA-T) are pre-assembled as T1, which converts the signal input into active Cas12a protein (Cas12a/gRNA-T/DNA complex). After the action of a self-reporting scgRNA, an amplified fluorescent signal and multiple active (decaged) gRNA molecules are output. T2 contains Cas12a protein and a probe for decaged gRNA, which can transduce the resulting decaged gRNA into another active Cas12a protein to produce an amplified fluorescent signal. scgRNA: switchable-caged guide RNA. **(C)** The protective oligonucleotide is designed to partially pair with the crRNA, thereby altering the conformation of the crRNA so that it cannot bind to the target DNA and inhibit the cleavage activity of Cas12a. The protective oligonucleotide breaks when exposed to ultraviolet (UV) light at 365 nm isolated from crRNA, thereby restoring the function of CRISPR-Cas12a detection. This system ensures that the target DNA is not reduced by Cas12a during the amplification phase, resulting in a stronger fluorescent signal.

In 2018, Jennifer and associates took advantage of trans-cleavage activity by Cas12a (formerly Cpf1) to establish a method named DETECTR (Chen et al., 2018), which achieves attomolar sensitivity for DNA detection (Figure 3A). The authors built a platform for testing different strains of human papillomavirus (HPV) after discovering that *Lachnospiraceae bacterium* ND2006 Cas12a (LbCas12a) can cut ssDNA in a robust, nonspecific manner. Human samples are first processed for recombinase polymerase amplification (RPA) reactions and subsequently incubated in a fluorophore quencher (FQ) reporter substrate ligated by ssDNA and Cas12a-crRNA, in anticipation of a readout of the fluorescent reporter signal. Due to the importance of point-of-care testing (POCT) (Drain et al., 2014), a rapid and visual detection method named Cas12a-based Visual Detection (Cas12aVDet) was proposed to further reduce the time to approximately half an hour based on DETECTR. It is a one-pot reaction with bright fluorescence directly visible under blue light (Wang et al., 2019).

Another Cas12a-based assay is called HOLMES (one-hour low-cost multipurpose highly efficient system) (Li et al., 2018b). It enables the efficient detection of SNP in human genotypes. HOLMES uses LbCas12a and polymerase chain reaction (PCR) for amplification and is applied to detect DNA viruses and RNA viruses with detection limits as low as 1–10 aM. The detection system has also been updated into a second version (HOLMESv2) (Li et al., 2019), which employs Cas12b (previously known as C2c1). Cas12b is another type V-B CRISPR-Cas system subtype that has a more preferred trans cleavage activity for dsDNA than Cas12a (Chen et al., 2018). HOLMESv2 combines target detection and loop-mediated isothermal amplification (LAMP) reactions, accomplishing both in one system, thus significantly increasing the efficiency, along with the capability of single-molecule testing like DNA, RNA, and even the degree of methylation of DNA. LAMP has the characteristics of high sensitivity, short reaction time, and no need for expensive reagents and specialized apparatus for clinical use (Notomi et al., 2000). Improving on the CRISPR-Cas12-based assay DECTECTR (Broughton et al., 2020), a two-step assay was optimized for identifying SARS-CoV-2 with faster detection and reasonable accuracy (95% correct positives and 100% correct negatives) using reverse transcription LAMP (RT-LAMP) for pre-amplification. A Cas12b-based one-pot method termed STOPCovid (SHERLOCK testing in one pot) also uses RT-LAMP amplification with proper Cas proteins from *Alicyclobacillus acidophilus* (AspCas12b) that can adapt to the corresponding temperature for detection (Joung et al., 2020). Another system using Cas12b is called iSCAN; version 2 simplifies the previous two-pot system to a single-pot system using the reverse transcription RPA (RT-RPA) method for amplification, with optimization of the solution system as well as the steps to perform clinical assays at optimal temperatures (Aman et al., 2022). Shi et al. (2021) developed a CRISPR-Cas–only amplification network (termed CONAN) which was supported by a CRISPR-Cas–powered catalytic nucleic acid circuit for ultrasensitive nucleic acid diagnostics (Figure 3B). In this study, switchable-caged guide RNA (scgRNA) with self-reporting capability was designed for single-nucleotide mutation (SM) detection in combination with Cas12a, which has PAM sequence recognition and trans cleavage activity. Its one-step reaction has a greater advantage in detecting hepatitis B virus (HBV) infection and human bladder cancer–associated SM.

A new Cas12a-based assay platform was recently developed to detect rabies virus (RABV) using the ECL method (Liu S. et al., 2022). The target molecule is amplified in the presence of two DNA probes, generating a large amount of ssDNA to activate the trans cleavage activity of CRISPR/Cas12a. Subsequently, single-stranded trigger (ST) strands are non-specifically degraded and inactivated, resulting in the inability to close DNA nanotweezers (DTs), i.e., hemin cannot be captured at the electrode, thus generating a high concentration and triggering an ECL signal.

In 2021, Chen and Ji’s team reported a class 2 type V-F CRISPR-Cas genome-editing system from *Acidibacillus sulfuroxidans* (AsCas12f1, 422 amino acids) (Wu et al., 2021), capable of targeting dsDNA with 5′T-rich protospacer adjacent motifs. They subsequently developed a Cas12f-based assay with low detection limits and detection concentrations higher than 500 pM without amplification (Wu Z. et al., 2022). However, the trans cleavage activity of Cas12f is inferior to Cas12a and Cas12b (Harrington et al., 2018), and further optimization of the system is needed to bring the CRISPR/Cas12-based biosensor to the clinic.

Many have started with the reaction solution to improve sensitivity in order to achieve this goal. In previous studies, Mg^2+^ in the buffer solution was found to be essential for the cis cleavage ability of Cas12a (Fonfara et al., 2016). A study by Wang et al. found its potential to affect trans cleavage activity as well (Lv H. et al., 2021). Studies on the effect of different metal cations on the cis and trans cleavage activity of LbCas12a have been carried out (Ma et al., 2020; Nguyen et al., 2020; Yue et al., 2021), for example, in a recent study, Mn^2+^ was found to have the ability to promote both cis and trans cleavage activity (Xie et al., 2022). Other properties of the buffer solution also contribute, such as high pH (8.5–8.6) solutions being more favorable for Cas12a to exert trans cleavage activity. Several components, such as polyethylene glycol (PEG), also significantly enhance the cleavage signal (Lv H. et al., 2021). Bovine serum albumin (BSA) and L-proline markedly improved the detection efficiency of Cas12a (Li Z. et al., 2021). In addition to the buffer solution, the ratio of Cas12a, crRNA, and target dsDNA at 1:2:2 also exhibited the maximum kinetic efficiency (Lv H. et al., 2021). Besides, it was found that the base profile of the ssDNA linked to the FQ-reporter also affects the nonspecific cleavage of Cas12a, with the Poly-C reporter having the highest efficiency for being cleaved, while the Poly-G reporter, in contrast, can barely be cleaved. An increase in the length of this sequence up to 8 promotes an enhancement in fluorescence intensity. Still, the increase is not significant after the peak of 8 is reached, and the synthesis of excessively long ssDNA also leads to an additional cost of detection (Lv H. et al., 2021). In a recent study, researchers found enhanced trans cleavage activity and significantly improved signal intensity by linking the fluorophore to the quencher with hairpin DNA (Rossetti et al., 2022). These studies set the first step toward continuous improvement of the reaction system in the future. Researchers have also optimized the assay by crRNA engineering, introducing DNA, elongating one end of the crRNA, and chemical modification of the crRNA, all reported to have improved the assay sensitivity (Park et al., 2018; Nguyen et al., 2020; Ooi et al., 2021). Besides, Yin et al. found that most suboptimal (suboptimal) sequences (e.g., VTTV, TCTV, and TTVV) have better performance relative to standard PAM (TTTV) by comparing several PAM sequences used for crRNA and devised a method named sPAMC (for suboptimal PAM of Cas12a-based test with enhanced flexibility, speed, sensitivity, and reproducibility) to achieve fluorescence analysis of viruses in a one-pot reaction with less than 20 min (Lu S. et al., 2022). Hu et al. designed a light-controlled one-pot assay ingeniously by designing a protective oligonucleotide to bind to crRNA which reduces the cleavage of Cas12a at the RPA stage, after that, the protective oligonucleotide is cut off by UV irradiation at 365 nm and crRNA is released for the readout of the signal (Figure 3C) (Hu M. et al., 2022). The photocontrolled crRNA reaction provides a stronger detection signal than the conventional one-pot detection method. To change the current situation of the lateral flow test (LFT)-DETECTR system, where the test zone signal is prone to false positives, a system called DIRECT (DNA-Immunoglobulin Reporter Endonuclease Cleavage Test) was designed using a composite probe consisting of the DNA part (biotinylated dsDNA connected to ssDNA with fluorescein) (FAM) and the antibody part (mouse anti-FAM IgG), that was capable of improving the signal readout of the test zone^2^, (Bhatt et al., 2022). A method called CLEVER (CRISPR-Cas12 integrated RT-LAMP Easy, Visual and Extraction-free RNA) makes detection faster and more convenient by optimizing the detection of N and E genes of SARS-CoV-2 by removing the RNA extraction step in the process of protein digestion (Bhatt et al., 2022). Cheng’s team performed pre-amplification of Cas12a assay by LCR (ligase chain reaction) to achieve ultra-sensitive visualization of microRNA detection. However, a specific measure may be limited to certain Cas enzymes and reaction systems to be effective, and further identification of reaction mechanisms still requires in-depth research (Qiu M. et al., 2022).

#### 2.3.3 Cas13-based biosensors and bioassays

In 2017, Zhang et al. initiated the groundbreaking use of Cas13a for highly sensitive detection (Figure 3A) by developing a SHERLOCK system targeting DNA or RNA (Gootenberg et al., 2017). The system first amplifies the target gene isothermally employing RPA or RT-RPA, a process that generates a large amount of DNA. Subsequently, T7 RNA polymerase comes into play and transcribes the DNA into RNA to serve as a substrate for Cas13a nuclease. The crRNA then complements this RNA. The technology was updated a year later with a second version (SHERLOCKv2) (Gootenberg et al., 2018). The new detection system achieved a 3.5-fold increase in signal sensitivity through the action of Csm6, a protein that activates collateral activity. In addition, SHERLOCKv2 also uses a lateral flow approach, thus providing rapid detection in areas lacking specialized equipment and promising to offer on-site diagnostic coverage in areas infected with viruses such as ZIKV and Gradenovirus (DENV).

To detect ZIKV and DENV directly in the bodily fluids, the researchers developed HUDSON (heating unextracted diagnostic samples to obliterate nucleases) (Myhrvold et al., 2018), allowing colorimetric reporting of signal readout. HUDSON has expanded the assay to include samples that can be pre-amplified with RPA, such as blood, plasma, serum, urine, and saliva, by lysing the viral particles with heat and chemical reduction to inactivate the ribonuclease resulting in the release of nucleic acid molecules. The combination of HUDSON and SHERLOCK reduces the time to obtain results to 1–2 h with limited sample and equipment requirements. To improve HUDSON, a sensing method with sensitivity and specificity termed SHINE (Streamlined Highlighting of Infections to Navigate Epidemics) was developed (Arizti-Sanz et al., 2020). In addition to shortening the detection time, the authors also developed a companion smartphone application that allows straightforward interpretation of the fluorescence signal in the test tube. The method was tested in 30 SARS-CoV-2 positive patient samples and 20 negative patient samples with 100% specificity for negative samples and 90% sensitivity for positive samples (27/30). HUDSON reportedly accelerated the extraction of viruses from nasal swabs and saliva samples during this process. The method reduced the LOD (limit of detection) by adding RNase H and optimizing the concentration of various components of the buffer solution. Another Cas13a-based assay achieves quantitative measurement of RNA without pre-amplification of the genome (Fozouni et al., 2021). The authors used a Cas13a homolog from *Leptotrichia buccalis* (Lbu), which is considered to have robust trans cleavage activity and sensitivity, followed by designing multiple combinations of crRNAs for use towards separate target gene regions of the virus, capable of detecting ∼100 copies/µl of SARS-CoV-2 virus. Subsequently, a mobile phone-based fluorescence microscope and reaction chamber was used to quantify the fluorescence signal for quantitative measurements.

Another promising assay platform called CREST (Cas13-based, rugged, equitable, scalable testing) was developed by Rauch et al. to tackle the issue of reagents, equipment, and cost that prevented the use of Cas13 assay systems on a large scale (Rauch et al., 2021). Rauch et al. developed Bluetooth-enabled, field-ready thermocyclers in combination with Taq enzymes to overcome the previous restriction of PCR amplification only in specialized laboratories. Then, the authors produced a P51 cardboard fluorescence visualizer powered by a 9-V battery so that the activity of detecting Cas13 could be visualized, and the results of the FQ-reporter could be read out. Although CREST has further reduced the assay cost from several angles, RNA extraction continues to rely on commercial kits. Therefore, another method called PEARL (precipitation-enhanced analyte retrieval) was proposed to cut down the reliance on commercial kits (Ponce-Rojas et al., 2021).

Cas13d ribonuclease is derived from *Ruminococcus flavefaciens* (CasRx). To expand CRISPR-based diagnostic strategies, researchers have used Cas13d for the first time in molecular diagnostics by developing a system for detecting SARS-CoV-2 called SENSR (Sensitive Enzymatic Nucleic-acid Sequence Reporter) as a modification of SHERLOCK (Brogan et al., 2020). The first step of SENSR is isothermal amplification, an initial 45-minute isothermal pre-amplification reaction by RT-RPA that generates a short dsDNA amplicon containing the T7 promoter sequence that is processed into the final RNA target sequence. The reported signal is read out by fluorescence or lateral flow, with the LOD determined by fluorescence readout to be ∼100 copies/µl and 92% (11/12) concordance with SENSR fluorescence analysis on lateral flow analysis. These measurements indicate its potential to be used in rapid diagnostic tests.

Achieving rapid detection of multiple pathogens has become a major issue owing to many human infectious diseases and the significant threat they pose to humans. This led one group of researchers to develop a CARMEN detection system (Combinatorial Arrayed Reactions for Multiplexed Evaluation of Nucleic acids), a scalable multi-pathogen detection platform with considerable statistical robustness (Ackerman et al., 2020). Researchers have tested it against 169 human-associated viruses, and a minimum of 10 published genomic sequences found it to have excellent sensitivity along with low cost. The method uses PCR or RT-RPA for amplification. The assay reaction system is performed in a conventional microtitre plate, and the droplets are sorted by 1,050 color codes of the solution. The system is a breakthrough in reducing the cost of the SHERLOCK assay, achieving simplification of steps, and reducing the diagnosis time in epidemic situations.

Nucleic acid amplification is an essential part of assay reporting signal enhancement, but the process can be subject to problems such as sample contamination and loss. For this reason, Zhou’s team developed a confinement effect-inspired Cas13a assay called the ultra-localized Cas13a assay, which elevates molecular concentrations in picoliter-sized droplets by droplet microfluidics for the assay (Tian et al., 2021). The fluorescence signal of the highly concentrated droplet system was reported to be quite marked, achieving RNA quantification at the single-molecule level. The new method was used to accurately count the cell-free microRNA and bacterial 16S rRNA in clinical serum samples while also allowing the diagnosis of the N gene for SARS-CoV-2. The results showed that 40 nasopharyngeal swab samples reported results in complete agreement with RT-PCR, demonstrating its power as a tool for quantitative molecular biology and accurate clinical diagnosis.

The CASCADE (CRISPR/CAS-based Colorimetric Nucleic Acid Detection) method was recently reported for the rapid and specific diagnosis of SARS-CoV-2 under naked eye conditions (López-Valls et al., 2022). Since colorimetric methods using AuNPs are not commonly used in Cas13-based detection systems, the authors chose the Cas13a enzyme to extend the detection tool, thus increasing its versatility. Cas13a enzyme system has the advantage that it lacks dependence on PAM sequences. The detection process of this system involves activating the trans cleavage ability of Cas13a, which leads to the shortening of ssRNA on the surface of AuNPs, causing their aggregation to produce color changes. The authors emphasize that the destabilizing aggregation of ssRNA-AuNPs is stronger than the previously reported effects based on Cas12a and ssDNA-AuNPs, and the naked eye quickly observes the changes. In the detection of targeted SARS-CoV-2, the method shows sensitivity at the level of picomolar concentrations, and its detection limit can be reduced to femtomolar (3 fM), and even attomolar (40 aM) ranges when combined with optimization of isothermal amplification. Remarkably short time (15–30 min), and the inexpensive visual readout suggest its ability for point-of-care testing of infectious diseases and the feasibility of future large-scale use. Besides, Hu F. et al. (2022) designed a one-pot system named ECS-CRISPR (Easy to operate, Contamination-free, and Stable CRISPR). The system consists of a nested inner and outer tube, and the CRISPR Cas13a fluorescent detection reagent is pre-existing in the inner tube. After the amplification reaction is completed, the product is transferred to the outer tube by centrifugation through hydrophobic wells. Fluorescent visual detection stimulation of the target nucleic acid is achieved under blue light-emitting diodes (LED), enabling the detection of pathogenic nucleic acids within 25 min without the need for particular instruments, thus effectively reducing contamination.

Jung et al. combined Cas13a with an electrochemical sensor to achieve nucleic acid-free amplification detection of SARS-CoV-2 (Heo et al., 2022). In this study, a redox probe conjugated with ssRNA is immobilized on the electrode surface modified with a nanocomposite (NC) and gold nanoflower (AuNF), and Cas13a trans cleavage ability is activated after digestion of reRNA to generate current. The sensor achieves detection limits as low as 4.4 × 10^−2^ fg/ml for ORF and 8.1 × 10^−2^ fg/ml for S genes with a dynamic range of 1.0 × 10^−1^–1.0 × 10^5^ fg/ml. Zhang et al. developed a new CRISPR Cas13a-gFET biosensor. In this system, the trans cleavage mechanism of CRISPR Cas13a is used to generate a current signal that can be combined with a graphene field-effect transistor (gFET) for amplification-free detection of nucleic acids with LODs down to the amolar range (Li et al., 2022d).

The above-mentioned methods for detecting nucleic acid analytes based on CRISPR are summarized in Table 1.

**TABLE 1:** Characteristics of CRISPR-based biosensors for molecular diagnosis.

System name	Effector	Preamplification	Readout	Sensitivity	Applications	Ref
NASBACC	Cas9	NASBA	Colorimetry	6.0 × 10^5^ copies/ml	Discrimination of different strains of Zika virus	Pardee et al. (2016)
—	Cas9	SDA and RCA	Colorimetry	2 pM (naked eyes)	Detection of the *Phytophthora infestans* genomic DNA	Chang et al. (2019)
—	dCas9	—	Colorimetry	NS	Detection of SARS-CoV-2, pH1N1, and pH1N1/H275Y viruses	Moon et al. (2020)
PC	dCas9	—	Fluorescence	5 × 10^−5^ nmol/ml	Detection of *Mycobacterium tuberculosis* DNA	Zhang et al. (2017)
CHP	Cas9	SDA and RCA	Fluorescence	aM	Detection of mutations in serum samples	Wang et al. (2021)
CRISPR-Chip	Cas9	—	Electrochemical	1.7 fM	Detection of gDNA from cell lines	Hajian et al. (2019)
—	Cas9	PER	Electrochemical	5 nM	Detection of SARS-CoV-2	Dai et al. (2020)
dCas9-ECL	dCas9	PCR	ECL	0.1 pg/μl	Detection of genome of *Listeria monocytogenes* and DNA in complex samples (e.g., milk and cell extracts)	Wu et al. (2022a)
DETECTR	Cas12a	RPA	Fluorescence	aM	Detection of HPV16 and HPV18 in human samples	Chen et al. (2018)
Cas12aVDet	Cas12a	RPA	Fluorescence	10 aM	Detection of mycoplasma	Wang et al. (2019)
HOLMES	Cas12a	PCR	Fluorescence	10 aM	Discrimination of SNP in human genotypes	Li et al. (2018a); Li et al. (2018b)
HOLMESv2	Cas12b	LAMP	Fluorescence	10 aM	Discrimination of SNP, detection of RNA and DNA methylation	Li et al. (2019)
STOPCovid	Cas12b	LAMP	Fluorescence or lateral flow	3.3 aM	Detection of SARS-CoV-2	Joung et al. (2020)
iSCANv2	Cas12b	RPA	Fluorescence	8 copies/µl	Detection of SARS-CoV-2	Aman et al. (2022)
—	Cas12a	BIIA	ECL	2.8 pM	Detection of RABV	Liu et al. (2022a)
—	Cas12f	—	Fluorescence	500 pM	—	Wu et al. (2022b)
DIRECT^2^	Cas12	PCR	Lateral flow	0.5 nM	Detection of the genome of *Dickeya solani*	Bhatt et al. (2022)
CLEVER	Cas12	LAMP	Lateral flow	NS	Detection of SARS-CoV-2	Bhatt et al. (2022)
SHERLOCK	Cas13	NASBA or RPA	Fluorescence	2 aM	Detection of viruses (ZIKV, DENV), bacteria, and SNPs; discrimination of virus strains	Gootenberg et al. (2017)
SHERLOCKv2	Cas13	RPA	Fluorescence and lateral flow	4.8 copies/ml	Detection of viruses (ZIKV, DENV), bacteria, and SNPs; discrimination of virus strains	Gootenberg et al. (2018)
HUDSON+						
SHERLOCK	Cas13a	RPA	Colorimetry	2 aM	Detection of viruses (ZIKV, DENV), bacteria and SNPs; discrimination of virus strains	Myhrvold et al. (2018)
SHINE	Cas13a	RPA	Colorimetry	10 copies/µl	Detection of SARS-CoV-2	Arizti-Sanz et al. (2020)
CREST	Cas13	PCR	Fluorescence	200 copies/µl	Detection of SARS-CoV-2	Rauch et al. (2021)
SENSR	Cas13d	RPA	Fluorescence and lateral flow	100 copies/µl	Detection of SARS-CoV-2	Brogan et al. (2020)
CARMEN	Cas13a	PCR or RPA	Fluorescence	0.9 aM	Detection of 169 viruses	Ackerman et al. (2020)
Ultralocalized Cas13a Assay	Cas13a	—	Fluorescence	0.1 fg/μl	Precisely counting microRNAs, 16S rRNAs, and SARS-CoV-2 RNA	Tian et al. (2021)
CASCADE	Cas13a	RPA or NASBA	Colorimetry	40 aM	Detection of SARS-CoV-2	López-Valls et al. (2022)
ECS-CRISPR	Cas13a	RPA	Fluorescence	3 copies/µl	Detection of ASFV	Hu et al. (2022a)
—	Cas13a	—	Electrochemical	4.4 × 10^−2^ fg/ml	Detection of SARS-CoV-2	Heo et al. (2022)
Cas14-DETECTR	Cas14	PCR	Fluorescence	6.0 × 10^3^ copies/ml	Detection of SNPs in human samples	Harrington et al. (2018)
—	Cas12a & Cas13a	RPA	Fluorescence	8 copies/µl	Dual-gene detection of SARS-CoV-2 (O and N gene) and ASFV	Tian et al. (2022)
Cas13a-gFET	Cas13a	—	Electrochemical	1 aM	Detection of SARS-CoV-2 and respiratory syncytial virus	Li et al. (2022d)

NS, not specified.

### 2.4 Non-nucleic-acid detection strategies

#### 2.4.1 Based on fDNA aptamers

The most crucial step of CRISPR-based detection strategies when analyzing non-nucleic acid target molecules is to find ways to convert the non-nucleic acid signal into a nucleic acid signal. Therefore, some detection strategies have designed receptors containing DNA molecules that have in common the release of DNA upon the addition of a foreign target analyte to the system to act as an activator of the CRISPR system, thus triggering the trans cleavage ability to send a signal *via* a fluorescent method.

**FIGURE 4:**
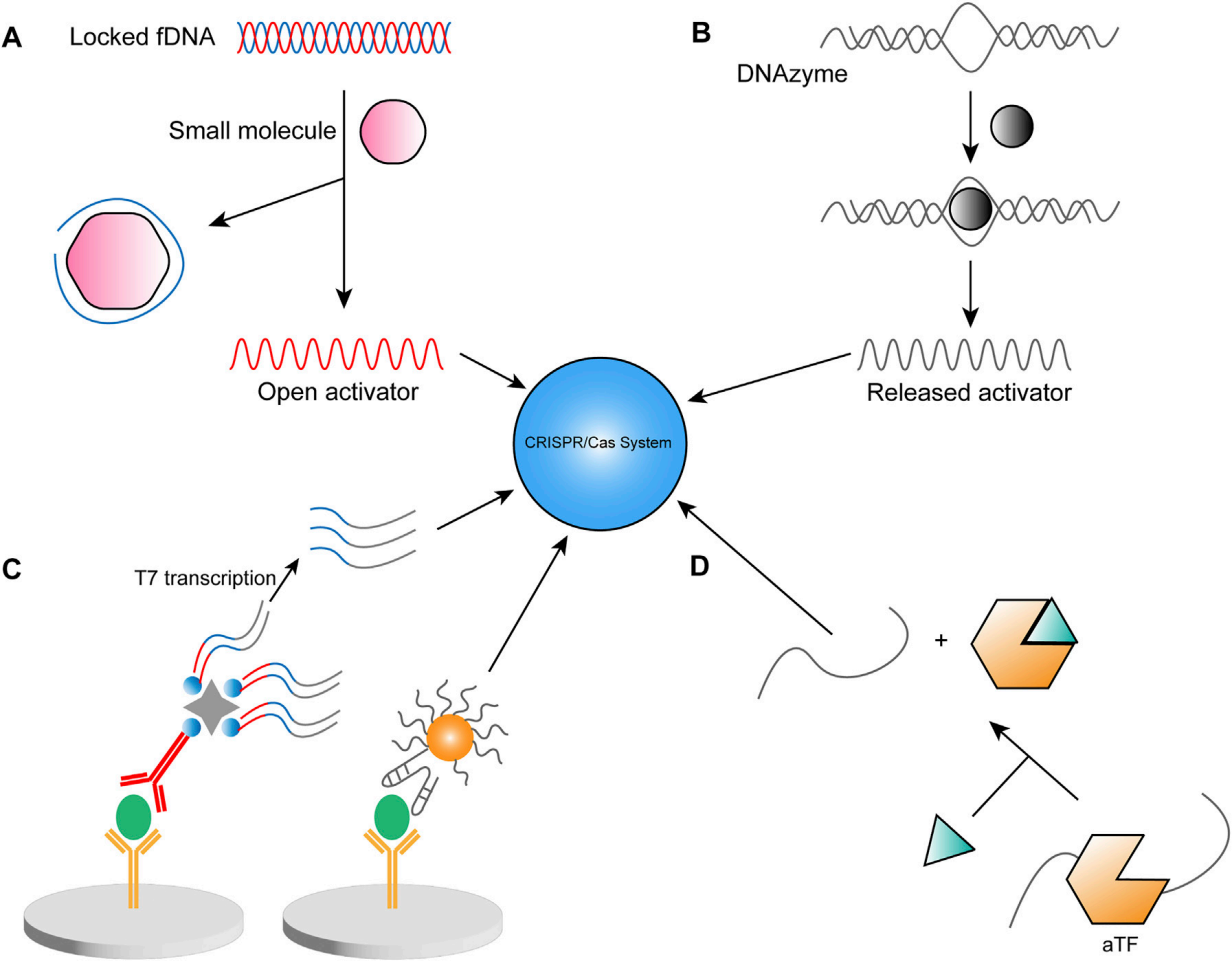
Strategies for CRISPR-based non-nucleic-acid detection. **(A)** Locked fDNA in the presence of a small molecule, one strand binds to it. It releases another complementary DNA strand to participate in the detection reaction **(B)** The DNAzyme starts in an inactive state and activates its cleavage ability with the involvement of specific ions, thus releasing short strands of DNA to participate in the detection system. **(C)** Left: Non-nucleic acid as antigen, linked to the CRISPR/Cas system and antibody (yellow)-antigen (green)-antibody (red) structure by a nucleic acid sequence containing the T7 promoter. Right: DNA-AuNP is used instead of antibody and is directly involved in the assay reaction. **(D)** Immobilized aTF-dsDNA complexes containing CRISPR target sequences change the conformation in the presence of aTF target small molecules, resulting in the release of dsDNA.

To address the lack of fluorescence intensity in complex samples, a novel luminescent material has been used in CRISPR detection, namely upconversion nanoparticle (UNCP) with high sensitivity and good resistance to environmental infections, and the trans cleavage ability of Cas12a allowing the departure of quenchers to trigger fluorescence (Li et al., 2020). Li et al. designed a polyacrylic acid-coated energy-confining UCNP with a sandwich structure (SUCNP), which was irradiated by a 980 nm portable laser to emit light, and the signal was able to be analyzed by a smartphone. The system cleverly utilizes fDNA, a DNA molecule complementary to crRNA, and a DNA aptamer that binds specifically to the target synthesized as a complex. When the target molecule is present, it competitively binds its aptamer, thus releasing DNA to activate Cas12a (Figure 4A). Similarly, Gao’s team constructed a UCNP-DNA-Fe_3_O_4_ probe to amplify fluorescent signals (Mao et al., 2022b). This biosensing approach can detect ochratoxin A, which appears in food and feed products. During the detection process, ochratoxin A binds to the aptamer and releases DNA to participate in subsequent reactions, achieving an overall detection time of approximately 1 h and a detection limit as low as 1.564 ng/ml in corn flour. In addition, Xiong et al. (2020) also designed a sensor using FQ-reporter by fDNA aptamer to detect ATP and Na^+^ rapidly. At ambient temperature (25°C), the method can complete the assay in two steps in less than 15 min and is suitable for on-site diagnosis or POCT. The other method for detecting ATP is different in that it is designed in such a way that crRNA is specifically recognized with the ATP aptamer so that in the presence of ATP, the trans cleavage ability of Cas12a is not activated due to the lack of the complementary strand of crRNA, and thus no fluorescent signal is generated (Peng et al., 2020).

The fDNA probes can also be combined with electrochemical approaches to achieve more accurate and sensitive detection. Lu et al. designed an inductively coupled plasma mass spectrometry (ICPMS)-based sensor. They selected Thulium (Tm) as the material for the reporter molecule because of the extremely low concentration and interference in the organism and the environment. After the trans cleavage event, Tm was captured in the supernatant by a streptavidin-coated magnetic bead (SA-MB) and subsequently monitored by ICPMS. The assay platform was used to analyze kanamycin residues, reducing the workflow to less than 30 min and obtaining detection limits as low as 4.06 pM (Hu et al., 2021). In response to the SARS-CoV-2 pandemic, an electrochemical sensor based on the viral nucleocapsid protein was designed (Han et al., 2022). A gold electrode surface was used as an electrochemical sensing interface with methylene blue labeled poly adenines DNA sequence immobilized on its surface as a signal reporter molecule. Np aptamers were hybridized with the activation strand as an arched probe to release the activator in the presence of a target molecule. The cleavage of the reporter molecule caused a reduction in the current of differential pulse voltammetry, lowering the LOD to 16.5 pg/ml within 30 min. The authors tested the detection potential in different complex samples such as tap water, milk, and serum, to explore the future industrialization of the sensor.

A small molecule modification strategy for fDNA has been reported in recent studies (Kim et al., 2021). Small molecules can serve as key components for the recognition of target proteins, and the presence or absence of complexes composed of target proteins and small molecules affects the binding efficiency of crRNA during downstream signaling. The eventual reduction in fluorescent signal due to target protein binding can help quantify the detection.

#### 2.4.2 Based on DNAzyme

Based on the selectivity of DNAzyme for metal ions, Wu’s team has developed a DNAzyme-based lead ion detection system for signal amplification *via* CRISPR (Chen et al., 2022). The method uses an enzyme consisting of two chains, the enzyme chain, and the substrate chain. When lead ions are present, the enzyme strand is energized to cut the substrate strand to release short ssDNA, activating Cas12a or Cas14a (Figure 4B). The authors verified the specificity of DNAzyme for the lead among various ions and purchased bottled water samples from the local area for testing, confirming its ability to detect actual water samples. The combination of CRISPR-Cas12a with DNAzyme achieved a detection limit as low as 0.48 nM for lead ions.

Although organophosphorus pesticides (OPs) have great potential to protect crops, their improper handling can lead to environmental pollution. A team of researchers has designed a dual enzyme assay combined with Cas12a to detect Ops (Fu et al., 2022). Briefly, the presence of OPs will affect the ability of acetylcholinesterase (AChE) to hydrolyze acetylthiocholine to thiocholine (TCh). In response, TCh can induce the degradation of MnO_2_ nanosheets and generate enough Mn^2+^ to stimulate the Mn^2+^-dependent DNAzyme activity. The method has been verified to possess strong resistance to interference in food matrices.

#### 2.4.3 Based on enzyme-linked immunosorbent assay

Enzyme-linked immunosorbent assay (ELISA) is a widely used assay technique in routine diagnostics. Zhou’s team developed a sensor called CLISA (the CRISPR/Cas13a signal amplification linked immunosorbent assay) to improve ELISA sensitivity using CRISPR/Cas13a for signal amplification (Chen et al., 2020). In this study, a primary antibody-target (antigen)-secondary antibody sandwich structure was established in which the T7 promoter, with the help of biotin and streptavidin, was recognized by T7 polymerase and initiated transcription, producing many copies of single-stranded RNA (Figure 4C). These RNAs were identified by Cas13a, which cleaved the reporter to produce fluorescence. The method has been validated for detecting an inflammatory factor, human interleukin-6 (human IL-6), and a tumor marker, human vascular endothelial growth factor (human VEGF), with LODs of 45.81 and 32.27 fg/ml, respectively, thereby achieving a hundredfold improvement in LOD. Subsequently, Song’s team designed Nano-CLISA by employing DNA-AuNPs as secondary antibodies to activate the trans cleavage ability of Cas12a (Figure 4C). The platform detects carcinoembryonic antigen (CEA) and prostate-specific antigen (PSA) biomarkers and achieves a 1000-fold higher sensitivity relative to ELISA, enabling quantitative analysis of proteins at the attomolar levels (Zhao et al., 2021). Similarly, an assay called CAFI (CRISPR/Cas12a assisted on-fiber immunosensor) was designed to examine small proteins in complex biological samples. The authors tested IFN-γ in human serum, sweat, saliva, and whole blood samples with detection limits as low as 58.8 aM (Deng et al., 2022).

#### 2.4.4 Based on allosteric regulation

In 2019, Zhang’s team used the recognition ability of allosteric transcription factors (aTF) to design a small molecule detection platform called CaT-SMelor (CRISPR-Cas12a- and aTF-mediated small molecule detector) (Liang et al., 2019). In this study, aTF has two structural domains, the DNA-binding structural domain, and the effector-binding structural domain. When the presence of small molecules initiates the metastable regulation of aTF, the binding ability of aTF to dsDNA is diminished, resulting in the release of the activation strand (Figure 4D). The aTF fused to the cellulose-binding domain (CBD-aTF) is immobilized on microcrystalline cellulose to analyze uric acid, a biomarker of chronic gout, in human blood samples. The fact that only 1 μl of blood is sufficient for analysis and that no sample preparation is required offer significant advantages for future detection of small molecules. A similar approach, SPRINT (SHERLOCK-based profiling of *in vitro* transcription), was devised to detect small molecules or enzymatic reaction products (Iwasaki and Batey, 2020). In these studies, aTF was used as a switch for transcription by RNA polymerase, just like a riboswitch, which activates the *in vitro* RNA polymerase transcription when a target molecule is present, and produces a large amount of RNA to provide an activator for Cas13a. SPRINT has been reported to have the ability to detect a wide range of small molecule compounds since aTF and riboswitch can have their specificities modified to target different target molecules. Chen et al. introduced Exo III, a nuclease that removes nucleotides, in their protocol to achieve the detection of TF (Li B. et al., 2021). When a TF called NF-κB p50 binds to dsDNA, its large spatial site block prevents the cleavage of Exo III from preserving dsDNA that can be used as an activator.

In addition to aTF, specific allosteric probes (APs) can also be used as a link in the signal transition. Usually, single-stranded DNA is used as the AP, containing a target recognition structural domain, i.e., a hairpin structure whose activity can be altered. In a detection system called APC-Cas (allosteric probe-initiated catalysis and CRISPR-Cas13a amplification reaction), the AP contains the aptamer domain of the pathogen, the primer binding domain, and the T7 promoter domain. The existence of the pathogen causes the AP hairpin structure to unfold to facilitate amplification of ssDNA into dsDNA, which subsequently initiates transcription to produce large amounts of ssRNA (Shen et al., 2020). Similarly, in the study by Li D. et al. (2022), AP was used for the recognition of tobramycin and contained an aptamer domain, a DNA amplification template domain, and an enzymatic cleavage site domain. Binding of tobramycin results in a conformational change of the AP to facilitate polymerase amplification and subsequent cleavage to provide substrate for the SDA reaction, which can perform a considerable amount of amplification to enable recognition of Cas12a.

PNKP (polynucleotide kinase/phosphatase) catalyzes the dephosphorylation of DNA 3′- and phosphorylation of DNA 5′- and plays a vital role in DNA repair in cells (Wang D.-X. et al., 2020). To achieve the detection of PNKP, a dual amplification sensing strategy was designed. The substrate DNA also includes a hairpin structural domain, a DNA amplification structural domain, and a nicking endonuclease (Nb.BbvCI) site structural domain. PNKP hydrolyzes the 3′-phosphate group to a hydroxyl group, amplified by DNA polymerase to initiate the SDA reaction.

Circulating tumor cells (CTCs) are tumor markers of great prognostic significance. In a recent study to detect CTCs, Yang’s team designed a CRISPR/Cas12a-based sensor (Lv Z. et al., 2021). In this study, a duplexed aptamer (DA) binds to a protein receptor on the cell surface upon encounter with CCRF-CEM cells, causing conformational reorganization and the release of an aptamer-complementary DNA strand (ACD) to the aptamer. Long ssDNA molecules with hundreds of repetitive aptamer units were constructed on MB by RCA reaction to regulate MDANs (Multivalent Duplexed-aptamer Networks). The CTC assay workflow is simple (two-step reaction) and fast (less than 60 min).

## 3 Summary and future directions

Since its discovery, CRISPR has been widely used in gene editing and biosensing and has excellent potential for clinical applications, especially in POCT. Currently, among all Cas enzyme types, the three most applied are Cas9, Cas12, Cas13, and their respective subtypes. Both the cis-cleavage ability of Cas9 and the trans-cleavage ability of Cas12 and Cas13 serve as the key to reporter signal amplification, with the hope that future researchers can discover or modify more Cas proteins with advantages. For example, in a recent study, Cas3 proteins from type I were also used to develop detection systems. Cas3-Operated Nucleic Acid detection (CONAN) was designed for targeted detection of nucleic acid molecules, based on the authors’ finding that EcoCas3 also has non-specific single-stranded DNA cleavage activity (Yoshimi et al., 2022). In one study, Cas12a and Cas13a were used in combination to improve orthogonal cleavage activity and multiplex detection. Interestingly, dual-gene amplified products from the multiplex RPA were simultaneously detected in a single tube (Tian et al., 2022). Future investigations on different Cas proteins and their mechanisms of action will help select the most appropriate protocols for other specific target molecules. In conclusion, the CRISPR/Cas system is an excellent tool for signal amplification, and its unique specificity advantage can play a significant role in testing.

Epidemic infectious diseases have always been a significant problem plaguing human progress. In particular, the COVID-19 pandemic is a massive challenge for many poorer countries lacking specialized equipment and laboratories. To address this crisis, developing biosensors with rapid, easy, and non-device-dependent detection is paramount. The CRISPR/Cas system has been integrated with biosensors, and many CRISPR-based detection platforms have emerged in the medical field. For example, the Omicron variant of SARS-CoV-2 was typed by designing different crRNAs (Liang et al., 2022), or some methods could test for various pathogens (Li C. et al., 2022; Lu P. et al., 2022; Liu X. P. et al., 2022; Qiu X. T. et al., 2022; Li F. A. et al., 2022; Huang et al., 2022; Shi et al., 2022; Wei et al., 2022; Zhang et al., 2022). Whether colorimetric, fluorescent, or electrochemical and electrochemiluminescence methods, CRISPR-based sensors are moving towards short and efficient detection, and even companion applications or smartphones have emerged, allowing diagnosis to be made at the comfort of one’s home. In addition to some of the classical detection strategies, systems have also arisen by engineering crRNA and combining it with PCR. Recently, a method called LEOPARD (leveraging engineered tracrRNAs and on-target DNAs for parallel RNA detection) was reported (Jiao et al., 2021), which describes its principle of detecting different RNA sequences with single nucleotide specificity by reprogramming tracrRNAs to bind desired cellular transcripts and finally forming Cas-crRNA complexes. This complex with intracellular RNA can target its complementary DNA and undergo cleavage, followed by a reporter signal by analyzing the size of the DNA. Wang and coworkers established a method named CRISPR/Cas9-typing PCR version 4.0 (ctPCR4.0), which simplifies the cumbersome steps of the previous version with ingredients by simply including the sample, Cas9-sgRNA, insert oligos, universal primer, and other PCR constituents. It is a one-pot assay that can be performed on a PCR only once and can be used to detect HPV DNA in cervical cancer cells with reasonable specificity and operability (Gao et al., 2021).

Detection of non-nucleic acid targets is an upcoming area where CRISPR and biosensing are combined. CRISPR is known to be a nucleic acid-sensitive detection system with specificity. Researchers must explore how to interpret the signals of small molecules, proteins, and other substances into nucleic acid signals. We still expect to see a detection system as good as SHERLOCK for large-scale commercial use in the future. Nevertheless, current research on non-nucleic acid targets is also of considerable interest. Many detection systems have developed, especially in the food and environmental fields, to detect pathogenic small molecules or parasites (Lei et al., 2022; You et al., 2022), or pathogens in agriculture (Jiao et al., 2022; Zhu et al., 2022).

Since the emergence of CRISPR technology in the Nobel Prize arena, increasing research and discoveries have been generated in this field. However, many issues remain in the application of CRISPR-based assays. For example, amplification of nucleic acid-like target molecules is still required for initial signal enhancement before Cas proteins cleave FQ-reporters to release fluorescence for signal amplification. Nevertheless, for some usage scenarios where rapid detection is needed, there is more potential to develop amplification-free approaches, and these detection systems should focus on improving test sensitivity. Next, for the wide range of biomarkers, from nucleic acid molecules to non-nucleic acid analytes there is still a need for more sensitive and specific detection systems to fill some of the gaps in the detection of target substances. More advanced and reliable assay strategies are being explored, from nucleic acid analytes to non-nucleic acid detectors. Future research will continue to improve detection strategies for both the amplification (-free) method and the CRISPR system. Newer Cas enzymes, more powerful readout systems, and more flexible ways of signal conversion and amplification will represent the possible directions of improvement. The prospect is enormous for artificial intelligence, novel materials, and engineering developments to empower CRISPR research.
