# Supplementary figures and images for: Research progress of CRISPR-based biosensors and bioassays for molecular diagnosis
Source: Front Bioeng Biotechnol. 2022 Sep 16;10:986233. doi: 10.3389/fbioe.2022.986233 (PMC9524266; doi:10.3389/fbioe.2022.986233)

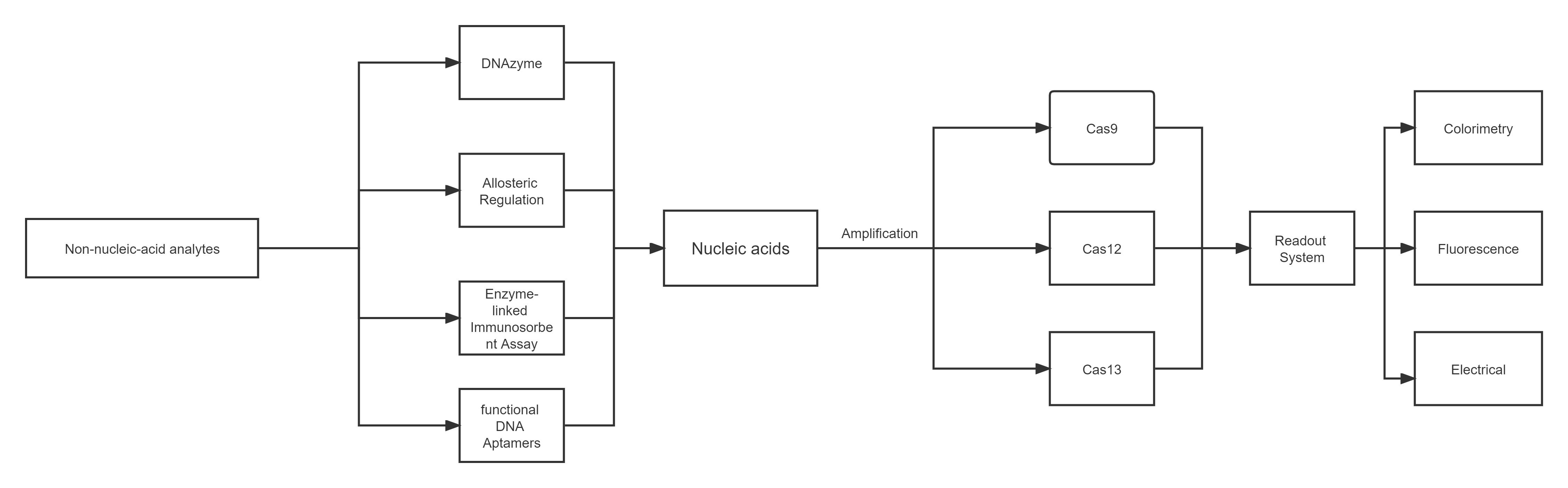

Supplement: Supplementary file 1 [file Image1.JPEG]
